# Supplementary material for: Differences in Leaf Morphology and Related Gene Expression between Diploid and Tetraploid Birch (Betula pendula)
Source: Int J Mol Sci. 2022 Oct 26;23(21):12966. doi: 10.3390/ijms232112966 (PMC9656147; doi:10.3390/ijms232112966)
Supplement: Supplementary file 1 [file ijms-23-12966-s001.zip › ijms-1970510-supplementary.pdf]

Supplementary Material

1 Supplementary Figures

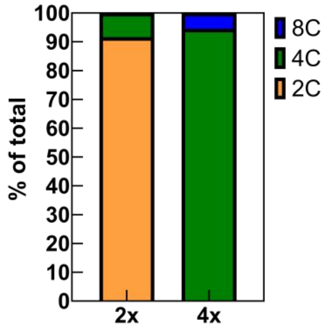

Supplementary Figure S1. Nuclear DNA ploidy distribution of diploid and tetraploid birches

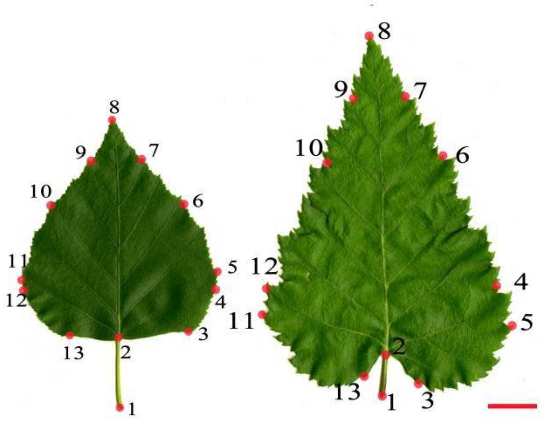

Supplementary Figure S2. Thirteen locations were used as landmarks in this study. Bar=2 cm

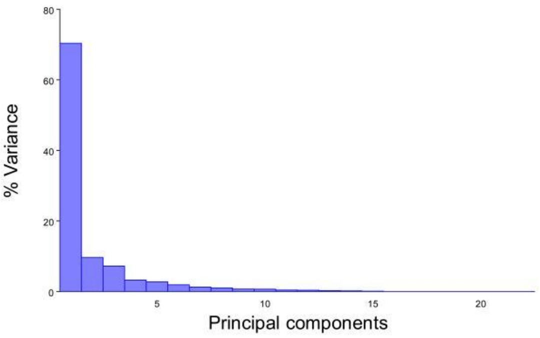

Supplementary Figure S3. Eigenvalues obtained from leaf principal component analysis (PCA)

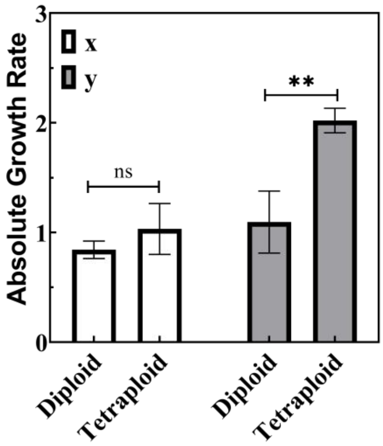

Supplementary Figure S4. Absolute growth rates of proximal (y) and distal (x) ends of diploid and tetraploid birches(n=3). Ns = not significant;**=P<0.01 (paired Student's t-test).

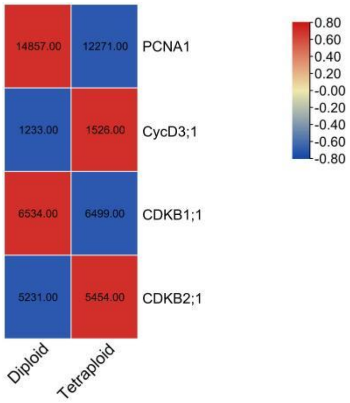

Supplementary Figure S5. Heat map of gene expression related to cell division

(A)

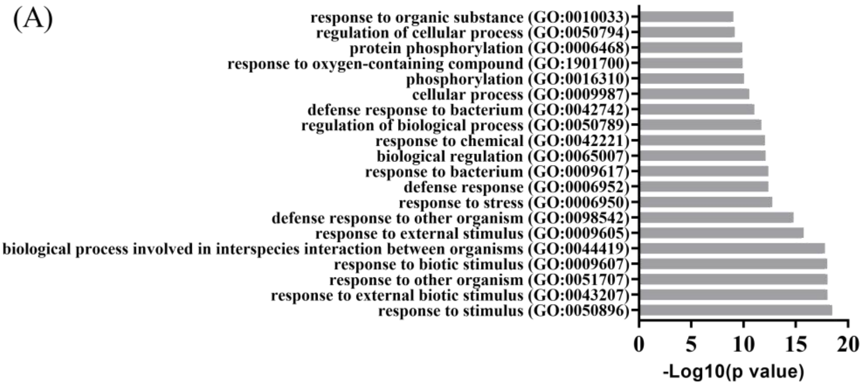

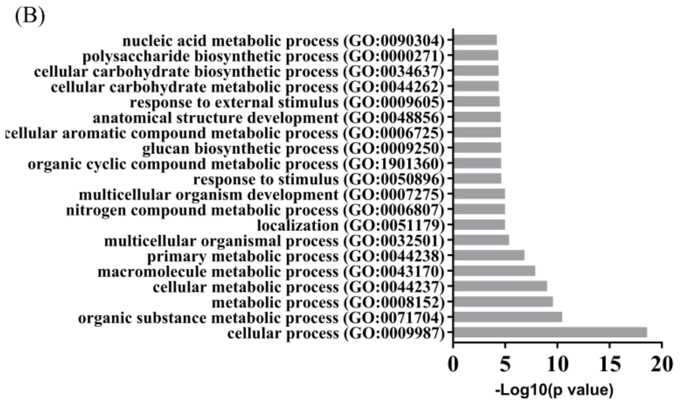

Supplementary Figure S6. GO (gene ontology) analysis of DEGs. 4x vs 2x up DEGs (A) and down DEGs (B) enriched GO analysis.

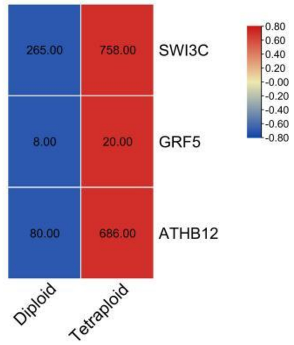

Supplementary Figure S7. Heat map of gene expression related to regulate leaf development

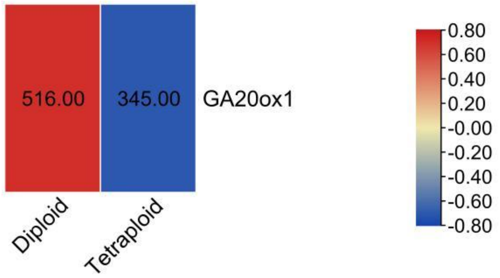

Supplementary Figure S8. Heat map of GA20ox1 gene expression

2 Supplementary Tables

Supplementary table S1 Gene ID and qRT-PCR prime sequences of genes used in the experiment

Gene Name	Gene ID	Upstream primers (5′-3′) Forward Primer	Downstream primers (5′-3′) Reverse Primer
CycD3;1	Bpev01.c0640.g0020	CCAAAGAGGGAGATACCCACG	AGCAGAGAACCCATAGTGCG
CDKB2;1	Bpev01.c0480.g0058	AGAAAGGTGGCGTCAGCAAT	GTCTTCTTGAGGGCCACGAT
CDKB1;1	Bpev01.c0224.g0013	TTCAGGTTGCTCGGTACTCC	AGGAGGTCAACACCATCAGG
PCNA1	Bpev01.c0383.g0019	CTCCTTTACCAAGGCGACCC	TGGGAGCCAAGTAGAACCGA
GRF5	Bpev01.c0051.g0114	GAGACACATGCACAGAGGGA	TGCTGAGTGGTTCTTGGTGA
BRI1	Bpev01.c1024.g0008	CGCTTGTGCATCGTTGGAAT	AAGGACCCAGGAAGCTGTTG
ATHB12	Bpev01.c0522.g0017	AGGAACAGCAAGACCAAGCG	GGCAGCAGACCAAGCTCATT
SWI3C	Bpev01.c0088.g0103	GCTCATGCCTCTGATTTGGACA	CCTCCCCTCTTGGAAGCAAT
18s	/	GAGGTAGCTTCGGGCGCAACT	GCAGGTTAGCGAAATGCGATAC
Actin	Bpev01.c1225.g0009	CATCTCTGATCGGAATGGAAG	AGATCCTTTCTGATATCCACG
GA20ox1	Bpev01.c0213.g0061	/	/
